# The role of junior enterprises in the development of human resources in biotechnology: a case report in Rio De Janeiro state

**DOI:** 10.1186/1753-6561-8-S4-P228

**Published:** 2014-10-01

**Authors:** Felipe Teixeira, Debora Moretti, Daniela Uziel

**Affiliations:** 1 Empresa Júnior Antônio Paes de Carvalho, Universidade Federal do Rio de Janeiro, Brazil

## Introduction

Junior Enterprises are nonprofit entities run by undergraduate students. The Junior Enterprise Antonio Paes de Carvalho (EJ-APC) was established in 2010 with the major goal of stimulate the entrepreneurial skills of students in the life sciences area by the development of biotechnological products and services. Since human resources trained in basic science at the university are poorly incorporated into the private sector, we aim to demonstrate that new technologies can be developed or new markets can be achieved by the students in order to stimulate their professional career out of the academic locus.

## Methods

Consists in the creation, establishment and management of the EJ-APC itself. The students incorporated as director can experience the daily work of an enterprise at all stages of its operation, from production to the presidency. Besides, students enrolled in products development can enhance their skills at the bench, using specific technologies.

## Results

Since its inception, it has had four directories, yearly elected, and for selection processes for new members. In 2010, there was one single project line (polyclonas antibodies), whereas in 2013 we already run 4 different lines: polyclonal antibodies, academic histologic slides, platinated materials and genotyping. A series of changes in the structure and organization of the company were driven by directors: i) the creation of a complex selection process; ii) greater participation in MEJ (Junior Enterprise Movement); iii) the establishment of strategic planning; and, iv) implementation of SOPs (Standard Operating Protocols).

## Conclusion

The EJ-APC has an icreasing number of students who engage in the management and implementation of projects, diversifying their professional perspective. Despite these advances sounds like an expected progression, we consider it an achievement in view of such strongly academic niche in which the EJ operates. At EJ-APC, students can learn and experience subject different from those in the traditional university teaching.

